# Suppression of SMOC2 alleviates myocardial fibrosis *via* the ILK/p38 pathway

**DOI:** 10.3389/fcvm.2022.951704

**Published:** 2023-03-02

**Authors:** Huang Rui, Fang Zhao, Lei Yuhua, Jiang Hong

**Affiliations:** ^1^Department of Cardiology, Renmin Hospital of Wuhan University, Wuhan, China; ^2^Cardiovascular Research Institute, Wuhan University, Wuhan, China; ^3^Hubei Key Laboratory of Cardiology, Wuhan, China; ^4^Department of Cardiology, The Central Hospital of Enshi Tujia and Miao Autonomous Prefecture, Enshi City, China

**Keywords:** SMOC2, cardiac fibrosis, cardiac remodeling, ILK, p38 MAPK

## Abstract

**Background:**

Fibrosis of the myocardium is one of the main pathological changes of adverse cardiac remodeling, which is associated with unsatisfactory outcomes in patients with heart disease. Further investigations into the precise molecular mechanisms of cardiac fibrosis are urgently required to seek alternative therapeutic strategies for individuals suffering from heart failure. SMOC2 has been shown to be essential to exert key pathophysiological roles in various physiological processes *in vivo*, possibly contributing to the pathogenesis of fibrosis. A study investigating the relationship between SMOC2 and myocardial fibrosis has yet to be conducted.

**Methods:**

Mice received a continuous ISO injection subcutaneously to induce cardiac fibrosis, and down-regulation of SMOC2 was achieved by adeno-associated virus-9 (AAV9)-mediated shRNA knockdown. Neonatal fibroblasts were separated and cultured *in vitro* with TGFβ to trigger fibrosis and infected with either sh-SMOC2 or sh-RNA as a control. The role and mechanisms of SMOC2 in myocardial fibrosis were further examined and analyzed.

**Results:**

SMOC2 knockdown partially reversed cardiac functional impairment and cardiac fibrosis *in vivo* after 21 consecutive days of ISO injection. We further demonstrated that targeting SMOC2 expression effectively slowed down the *trans*-differentiation and collagen deposition of cardiac fibroblasts stimulated by TGFβ. Mechanistically, targeting SMOC2 expression inhibited the induction of ILK and p38 *in vivo* and *in vitro*, and ILK overexpression increased p38 phosphorylation activity and compromised the protective effects of sh-SMOC2-mediated cardiac fibrosis.

**Conclusion:**

Therapeutic SMOC2 silencing alleviated cardiac fibrosis through inhibition of the ILK/p38 signaling, providing a preventative and control strategy for cardiac remodeling management in clinical practice.

## Introduction

Adverse cardiac remodeling, defined as changes in left ventricular (LV) anatomical structure and function, is a common complication developed in the diseased heart as a result of an array of intra- and extra cardiac pathophysiological conditions and is correlated with a poor outcome and shortened survival in hospitalized individuals presenting with heart conditions (1–3). Implications from substantial evidence suggest that abnormal hypertrophy of existing cardiomyocytes and perpetual activation of cardiac fibroblasts are fundamental pathomechanisms of heart failure (4). Previous studies have demonstrated the involvement of AKT and MAPK pathways in myocardial remodeling and myocardial fibrosis, which has been widely recognized (5, 6). However, fibrosis of the myocardium is a complex pathological process with a multifactorial etiology, and further investigation into the specific in-depth molecular mechanisms is still imperative for improving prognoses (4, 7).

SMOC2, SPARC-related modular calcium-binding protein 2, which encodes a secreted modular protein containing a pair of FS domains and an EC domain and predominantly resides in the kidneys, lungs, myocardium, skeletal muscles, and ovaries, forms part of the BM-40/SPARC/osteonectin protein family as SMOC1 (8, 9). As a result of binding to cell surface receptors, cytokines, and proteases, SMOC2 regulates the cell-matrix interaction, facilitating ischemic myocardial microvascular regeneration and tumor cell growth (8, 10–12). SMOC2 is implicated in some preclinical studies as contributing to the regulation of fibrotic disorders (13–16). Study results previously suggested that SMOC2 could stimulate renal fibroblast-to-myofibroblast conversion or transition, facilitate extracellular matrix synthesis, and ultimately result in kidney fibrosis (13). As evidence for this finding, Xin et al. (14) disclosed that silencing SMOC2 could affect renal inflammation, fibrosis, and function in chronic kidney disease (CKD) mice. A subsequent study discovered that SMOC2 knockout was available to mitigate bleomycin-induced pulmonary fibrosis (15). In addition, a network analysis of expression profile data has identified that SMOC2 may be implicated in the pathogenesis of heart failure (17), which may allude to a possible connection between SMOC2 and cardiac fibrosis despite the lack of studies demonstrating this link.

Studies have shown that SMOC2 exerts its physiological effects *in vivo* in part by transducing integrin-linked kinase (ILK)-mediated intracellular signaling cascades (18, 19). SMOC2 is also involved in activating ILK directly, enabling it to propel the cell cycle forward (20). Overexpression of ILK increases the collagen type I expression in cardiac fibroblasts by activating nuclear factor-κB (NF-κB) while silencing ILK resulted in the reverse effect (21). Additionally, p38-MAPK, a generally accepted pathway involving fibrosis, can also be activated by ILK (22–24). Researchers found that ILK directly activated P38 MAPK to influence osteoblading effects, which could be reversed if ILK was targeted to be silenced (24).

As yet, it is unclear if ILK contributes to cardiac fibrosis by stimulating p38. Consequently, we designed and conceived this study to investigate the effect of SMOC2 on cardiac fibrosis and to understand the detailed mechanisms involved.

## Materials and methods

### Reagents

Transforming growth factor-β (TGF-β, 763104) was purchased from Biolegend (San Diego, CA, USA). Isoprenaline (ISO, I5627) was obtained from Sigma-Aldrich and prepared in DD water. The SMOC2 (SC-376104, 1:500 for western blot and 1:100 for satining) and ILK (SC-20019, 1:500) antibodies were obtained from Santa Cruz Biotechnology Inc. (Santa Cruz, CA, USA). Antibodies against collagen type I (Col I, 1:1000), collagen type III (Col III, 1:1000), phospho-JNK (Tyr185,1:1000), JNK (1:2000), phospho-p38 MAPK (Thr180/Tyr182,1:1000), t-p38 (1:1000), phospho-AKT (1:5000), t-AKT (1:5000) and glyceraldehyde 3-phosphate dehydrogenase (GAPDH,1:4000) were obtained from Proteintech (Wuhan, China). Anti-α smooth muscle actin (a-SMA, 1:100 for staining) antibody was purchased from Abcam (Cambridge, UK).

### Animals and treatments

All animal experimental procedures were carried out under the supervision of the Animal Care and Use Committee of Renmin Hospital of Wuhan University and in compliance with established guidelines published by the National Institutes of Health of United States. Eight-week-old male C57BL/6 mice (body weight 23.5 ± 2 g) were provided by the Experimental Animal Center of the Three Gorges University (Hubei, China) for this study, and were acclimated to the experimental scenarios for 5–7 days before they were used. Under standard environmental conditions, all animals were fed a pellet diet and had access to water without restrictions. Mice were subcutaneously injected with ISO (10 mg/kg for 3 days and 5 mg/kg for 11 days) to induce cardiac fibrosis (25–27). For the control group, an equal volume of normal saline solution was administered. Four weeks before ISO injection, mice received a single intravenous injection of adeno-associated virus 9 (AAV9) carrying small hairpin RNA against SMOC2 (sh-SMOC2) and its corresponding negative control (shRNA) generated by Obio technology (Shanghai, China) to verify its role *in vivo*. Animals were randomly assigned to four groups: Control + sh-RNA, Control + sh-SMOC2, ISO + sh-RNA, and ISO + sh-SMOC2. Animals were sacrificed after an excessive inhalation of CO_2_, having their hearts and tibias harvested and measured to calculate the heart weight (HW)/body weight (BW) and HW/tibia length (TL).

### Echocardiography

Following modeling, echocardiographic evaluations were conducted under light anesthesia with a Vinno 6th ultrasound device with Doppler imaging (VINNO6, Vinno Corporation, China). At the level of the left ventricular short-axis papillary muscle, M-mode tracking was used to measure the following parameters: left ventricular end-systolic diameter (LVEDs), left ventricular end-diastolic diameter (LVEDd), left ventricular posterior wall thickness (LVPW), interventricular septal thickness (IST), ejection fraction (EF), and left fractional ventricular shortening (FS).

### Cell culture and treatment

A mixture of trypsin and collagenase II was used to extract CFs from the hearts of 1–3 days old mice. CFs were cultured in a medium with 10% FBS (fetal bovine serum) at 37°C with 5% CO2 after removing non-adherent cells after 1 h. Non-adherent cells were removed after 1 h, and the adherent CFs were cultured in DMEM/F12 medium containing 10% FBS (fetal bovine serum) at 37°C with 5% CO2. The following experiments were conducted with 2–3 passages of CFs. Following 8 h of serum-free DMEM/F12 culture, cells were stimulated with TGFβ. In order to knock down the SMOC2, CFs were transfected with short hairpin (sh)RNA adenovirus against SMOC2 performed by Shanghai Jikai Gene Chemical Technology Co., Ltd. As a negative control, adenovirus expressing short hairpin (sh)GFP was used. To confirm the role of ILK, CFs were transfected with adenovirus carrying ILK (Ad-ILK) or green fluorescent protein (Ad-GFP).

### Determination of collagen contents

Commercial ELISA kits (collagen I and collagen III ELISA kits from Uscnlife, Wuhan, China) were used to measure collagen I and III content in the cell culture supernatant after cardiac fibroblasts were incubated with or without TGFβ.

### Histological analysis and immunofluorescence

Heart tissue samples were fixed in 4% paraformaldehyde and embedded in paraffin before being cut into 5-micron sections. Heart tissue morphology and cardiac fibrosis were assessed with hematoxylin and eosin (HE) and Masson staining, respectively. Quantitative analysis was performed using Image J software.

CFs were stained with immunofluorescence in a well-cultured condition. Briefly, cell permeabilization was enhanced after fixation with 4% paraformaldehyde for 20 min, followed by incubation with 50–100 μl of disruption buffer for 10 min at room temperature. Cell coverslips were incubated with anti-α-SMA and anti-SMOC2 overnight at 4°C. The next day, cells were incubated with goat anti-mouse (SMOC2) pre-adsorbed or goat anti-rabbit (α-SMA) pre-adsorbed secondary antibodies for 1 h. Nuclei were subsequently stained with 4′,6-diamino-2-phenylindole (DAPI) and images were taken with a fluorescence microscope.

### Western blot and RT-PCR

The heart tissues or cells were prepared to extract total proteins with RIPA lysis buffer (Servicebio, Wuhan, China). The extracts were resolved *via* 8 and 12% sodium dodecyl sulfate-polyacrylamide gel electrophoresis (SDS-PAGE) and transferred to PVDF membranes (Millipore Corp. Bedford, MA, USA) using standard protocols. And then, the membranes were incubated with skim milk at room temperature for 1 h. Membranes were incubated with specific primary antibodies at 4°C overnight, followed by bio-tinylated secondary antibodies (goat anti-mouse HRP and goat anti-rabbit HRP) for 2 h at room temperature. The membranes were washed with TBST three times between each step mentioned above. Finally, the blots were detected by a hypersensitive ECL reagent (Biosharp, Beijing, China).

Total RNA was extracted using TRIzol reagent (Servicebio, Wuhan, China), and a cDNA synthesis kit was carried out to synthesize cDNA following the manufacturer’s protocol. Real-time PCR was carried out using EnTurboTM SYBR Green PCR SuperMix Kit (ELK Biotechnology). The mRNA levels were normalized to GAPDH. Molecular biological tests were conducted by individuals who were unaware of the treatment conditions of the animals and cells.

### Statistics analysis

SPSS 26.0 software was used for analysis. All results are presented as mean ± standard error of the mean, and *t*-test was used for comparison between two groups, and one-way ANOVA was used for three or more groups, followed by post L-S-D test. Differences were considered statistically significant with *p* < 0.05.

## Results

### SMOC2 was highly expressed in ISO-induced mouse model

**FIGURE 1:**
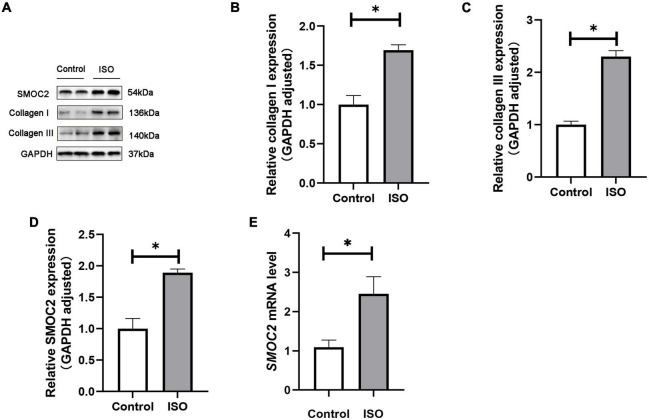
SMOC2 expression was increased in ISO-induced mice hearts. **(A–D)** Western blot image and quantitative results of the SMOC2, collagen I, and collagen III in the control group and the ISO group (*n* = 6). **(E)** RT-PCR analysis of SMOC2 mRNA levels in the two groups (*n* = 6). *p < 0.05.

We first investigated whether SMOC2 expression was altered in ISO-induced mouse model. Mice were treated daily with ISO or an equal volume of saline as control for 21 days as described. It was found that SMOC2 protein levels increased markedly in the ISO group, as was the accumulation of collagen types I and III (Figures 1A–D). Notably, RT-PCR further revealed increased expression level of SMOC2 in the ISO group (Figure 1E). The above observations strongly implicate that SMOC2 may play a critical role in regulating ISO-induced myocardial remodeling.

### SMOC2 knockdown partially reversed the myocardial function loss and myocardial fibrosis in response to ISO stimulation *in vivo*

We next sought to explore the specific effects of SMOC2 in ISO-induced cardiac fibrosis. Considering the up-regulation of SMOC2 expression in the ISO-induced mouse model, we constructed a SMOC2-knockdown AAV9 system to infect mice as previously described.

**FIGURE 2:**
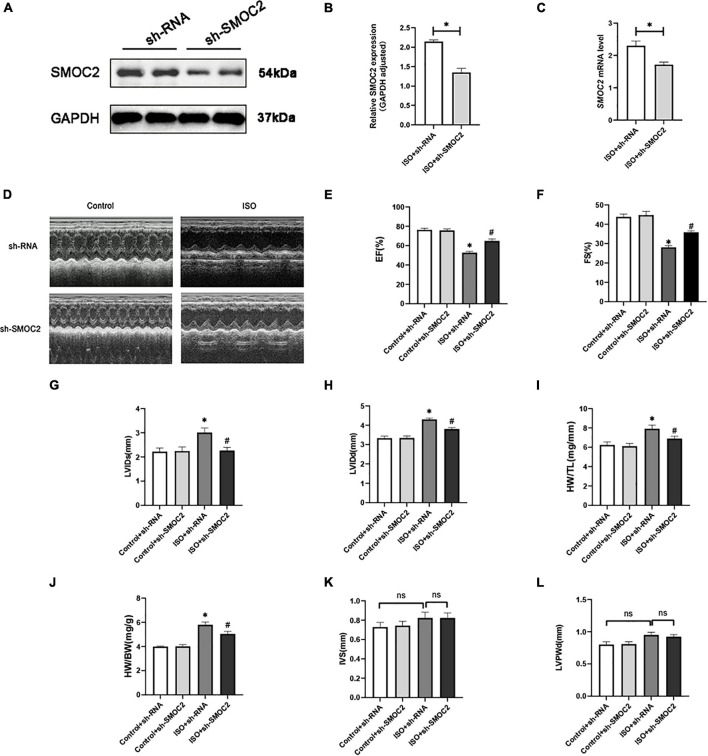
SMOC2 knockdown partially reversed the myocardial function loss. **(A,B)** SMOC2 expression after sh-RNA or sh-SMOC2 injection in mice heart (*n* = 6). **(C)** mRNA levels of SMOC2 after sh-RNA or sh-SMOC2 injection in mice heart (*n* = 6). **(D)** Representative images of echocardiographic measurement of cardiac function in mice. **(E–L)** Echocardiographic measurements of ejection fraction (EF), fractional shortening (FS), left ventricular internal diameter at end-systole (LVIDs), left ventricular internal diameter at end-diastole (LVIDd), HW/TL, HW/BW, interventricular septal thickness at diastole (IVS), and left ventricular posterior wall thickness at diastole (*n* = 8). *p < 0.05 vs. the control + sh-RNA group. ^#^*p* < 0.05 vs. the ISO + sh-RNA group. n.s., non-significant.

As shown in Figures 2A–C, hearts of animals administered sh-SMOC2 showed a significant decrease in SMOC2 mRNA and protein expression after 4 weeks compared to hearts of mice treated with sh-RNA. Mice infected with sh-RNA subjected to ISO injection for 3 weeks developed cardiac hypofunction, manifested as a decrease in EF, FS, and elevation in LVEDd, LVEDs, HW/TL, and HW/BW (Figures 2D–J). Notably, cardiac function was partially reversed in mice injected with sh-SMOC2 (Figures 2D–J). Neither ISO administration nor infection with sh-SMOC2 affected ventricular wall thickness, however (Figures 2K, L).

**FIGURE 3:**
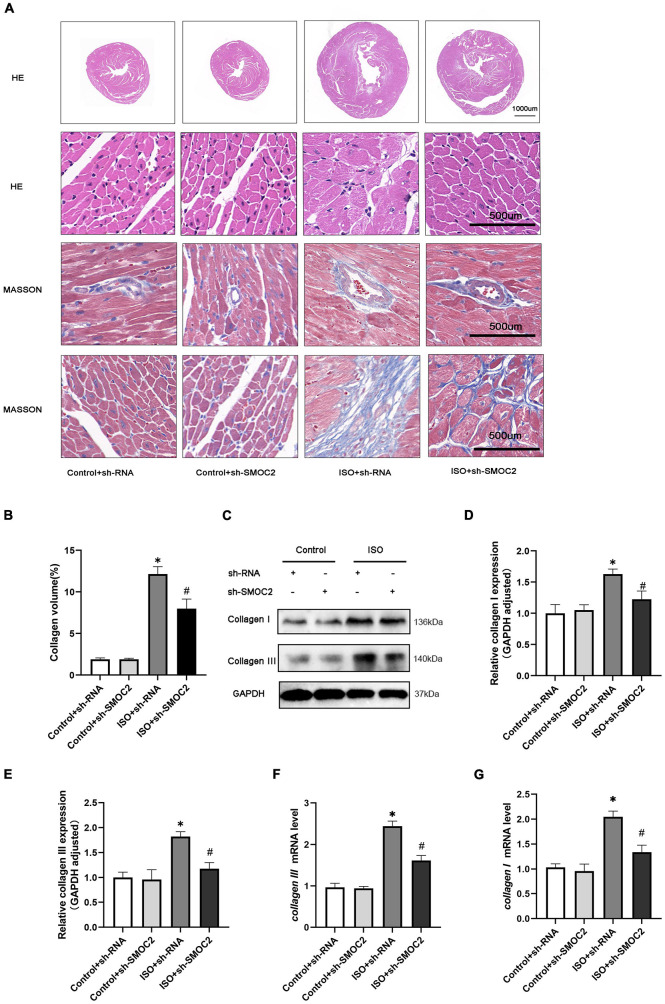
SMOC2 knockdown partially reversed the myocardial fibrosis in response to ISO Stimulation *in vivo*. **(A,B)** Representative images of HE and Masson staining and quantified analysis (*n* = 6). **(C–E)** Representative images of Western blots and the quantitative data (*n* = 6). **(F,G)** The relative mRNA levels of Collagen I, Collagen III (*n* = 3). *p < 0.05 vs. the control + sh-RNA group. ^#^*p* < 0.05 vs. the ISO + sh-RNA group. n.s., non-significant.

In addition, morphological examination revealed that mice experienced more severe myofibrillar disarrangement and fibrosis after 3 weeks of ISO injection (Figures 3A, B). Moreover, these histological changes weakened after SMOC2 deficiency (Figures 3A, B). To substantiate this further, we detected biomarkers of myocardial fibrosis. As expected, collagen I and collagen III protein and mRNA levels were down-regulated post-sh-SMOC2 injection (Figures 3C–G). These findings implied an underlying physiopathological impact of SMOC2 in modulating cardiac fibrosis, and SMOC2 knockdown in mice can partially reverse the myocardial function reduction and myocardial fibrosis in response to ISO stimulation *in vivo*.

### Targeting SMOC2 expression attenuates myocardial fibrosis *via* the ILK and p38 pathway

**FIGURE 4:**
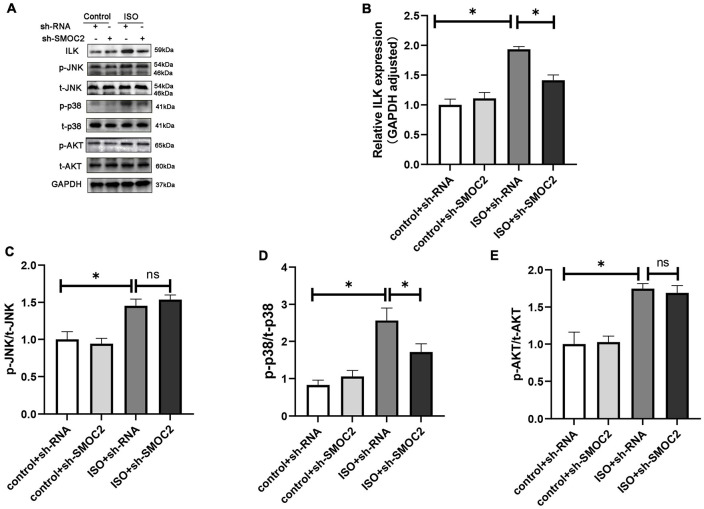
Targeting SMOC2 expression attenuates myocardial fibrosis *via* the ILK/p38 pathway. **(A–E)** Representative Western blots and quantitative results of ILK, t-AKT, p-AKT, t-JNK, p-JNK, t-p38, and p-p38 (*n* = 6). *p < 0.05. n.s., non-significant.

Next, we went on to achieve further insight into the potential mechanisms accounting for our previous findings. Pre-existing research has identified that SMOC2 could activate downstream ILK (20, 28), which was reported to be involved in the fibrotic pathological process (21). Consequently, we focused on the expression of ILK. It was revealed that, compared with the control group, the ISO + shRNA injection group had a significantly higher ILK level, and, importantly, the effect of ILK production can be relieved by SMOC2 knockdown (Figures 4A, B). Given that the AKT and AMPK pathways have been implicated in the pathophysiology of fibrosis, we examined whether these pathways are involved in the process of SMOC2-medicated myocardial fibrosis. It was found that p-AKT, p-p38-MAPK, and p-JNK-MAPK were significantly elevated in the ISO + sh-RNA group, while the p-p38 was decreased in the ISO + sh-SMOC2 group (Figures 4A, D). Strikingly, knocking down SMOC2 did not affect the expression of p-JNK and p-AKT (Figures 4C, E and Supplementary Figures 1A–F). Collectively, these findings demonstrate that the ILK and p38 pathway is likely to participate in the ISO-induced cardiac fibrotic process *in vivo*.

### SMOC2 is up-regulated in TGFβ-induced cardiac fibroblasts

**FIGURE 5:**
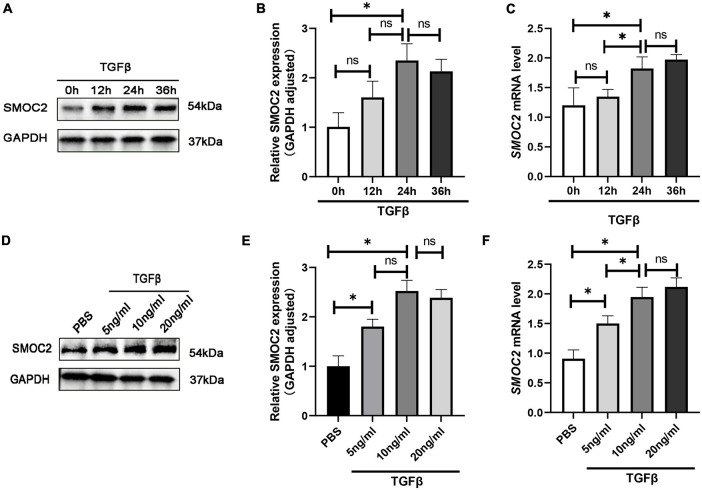
SMOC2 is up-regulated in TGFβ-induced cardiac fibroblasts. **(A,B)** Representative Western blots and quantitative results of SMOC2 in cardiac fibroblasts induced by different concentrations of TGFβ (*n* = 6). **(C)** mRNA levels of SMOC2 in cardiac fibroblasts induced by different concentrations of TGFβ (*n* = 3). **(D,E)** Representative Western blots and quantitative results of SMOC2 after induction of TGFβ at different times (*n* = 6). **(F)** mRNA levels of SMOC2 in cardiac fibroblasts after induction of TGFβ at different times (*n* = 3). *p < 0.05. n.s., non-significant.

To gain further insight into the regulation of SMOC2 in cardiac fibrosis, we conducted *in vitro* experiments subsequently. Neonatal myocardial fibroblasts were separated by a differential adherent as described to explore the role of SMOC2 in TGFβ-induced fibroblast activation. The results showed TGFβ induced fibroblasts activation in a dose-dependent and time-dependent manner. After being treated with TGF β (10 ng/ml) for 24 h, the protein concentrations of SMOC2 reached the highest (Figures 5A–F). Consequently, the cells were treated with TGFβ at 10 ng/ml for 24 h before being harvested in subsequent analysis.

### Knocking SMOC2 improves the proliferation, migration, differentiation and exacerbates fibrosis of fibroblasts *in vitro*

**FIGURE 6:**
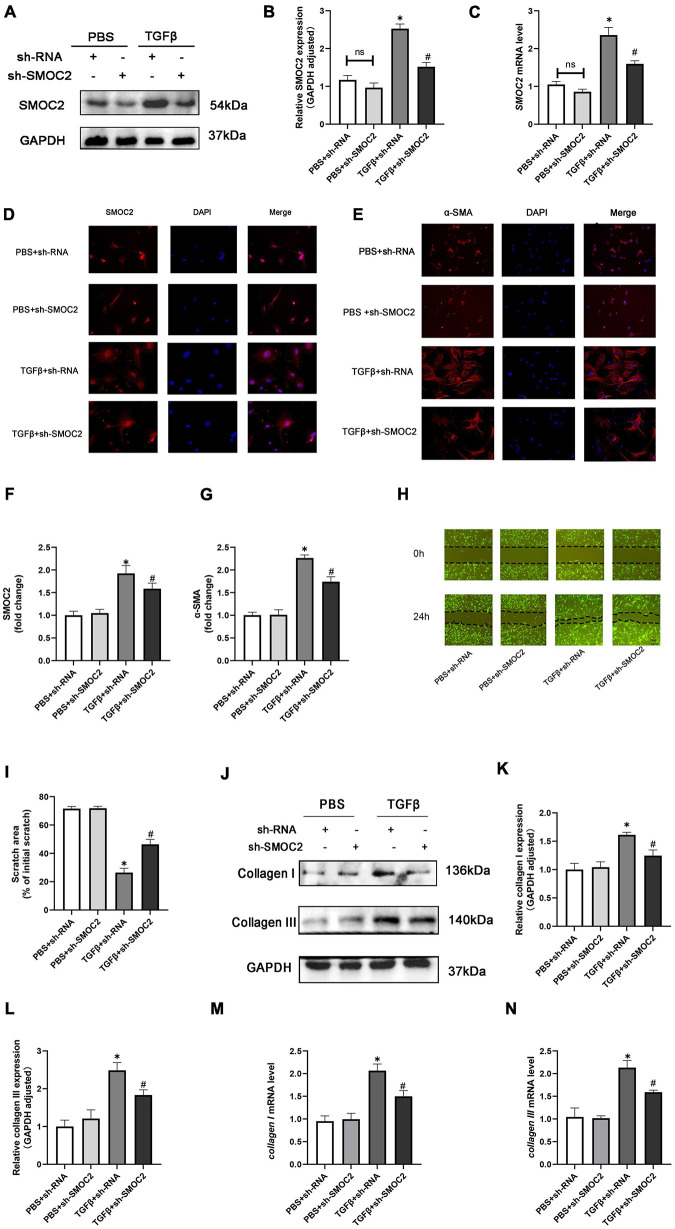
Targeting SMOC2 expression improves the proliferation, migration, and differentiation and exacerbates fibrosis of fibroblasts *in vitro*. **(A,B)** SMOC2 expression after sh-RNA or sh-SMOC2 infection (*n* = 6). **(C)** mRNA levels of SMOC2 after sh-RNA or sh-SMOC2 injection (*n* = 6). **(D–G)** Immunofluorescence staining of SMOC2 and α-SMA for the indicated groups (*n* = 6). **(H,I)** Representative images of the wound scratch assay at 0 and 24 h (*n* = 5). **(J–N)** Collagen I and collagen III expressions after sh-RNA or sh-SMOC2 infection in cardiac fibroblasts (*n* = 6). *p < 0.05 vs. the control + sh-RNA group. ^#^*p* < 0.05 vs. the TGF β + sh-RNA group. n.s., non-significant.

In order to discuss the potential effects and mechanisms underlying the pro-fibrotic effect of SMOC2, we infected CFs with sh-SMOC2 or sh-RNA as control. As shown in Figures 6A–C, after being treated with TGFβ for 24 h, SMOC2 protein and mRNA levels increased. However, these changes could be relieved post sh-SMOC2 infection. Furthermore, these findings were confirmed by an immunofluorescence stain for SMOC2 (Figures 6D, F).

The up-regulation of α-SMA expression, as a hallmark marker for differentiated myofibroblasts, was examined using immunofluorescence staining. As anticipated, the level of α-SMA was elevated in TGFβ + shRNA group, while SMOC2 down-regulation reduced the level of α-SMA (Figures 6E, G). Further analysis revealed that knocking SMOC2 could decrease the proliferation of TGFβ-induced CFs, as indicated by wound-healing scratch experiments (Figures 6H, I). Further analysis implicated that the protein and mRNA levels of collagen I and collagen III increased in the TGFβ + shRNA group, while knockdown of SMOC2 expression significantly inhibited type I and type III collagen production (Figures 6J–N).

Following that, we measured the collagen production of fibroblasts in the media. In agreement with the results above, the protein levels of fibrotic biomarkers such as type I and Type III collagen in the medium were elevated in the TGFβ group, whereas SMOC2 knockdown could relieve these changes (Supplementary Figures 2A, B).

### Targeting SMOC2 expression ameliorates fibrosis *via* the ILK/p38 pathway *in vitro*

**FIGURE 7:**
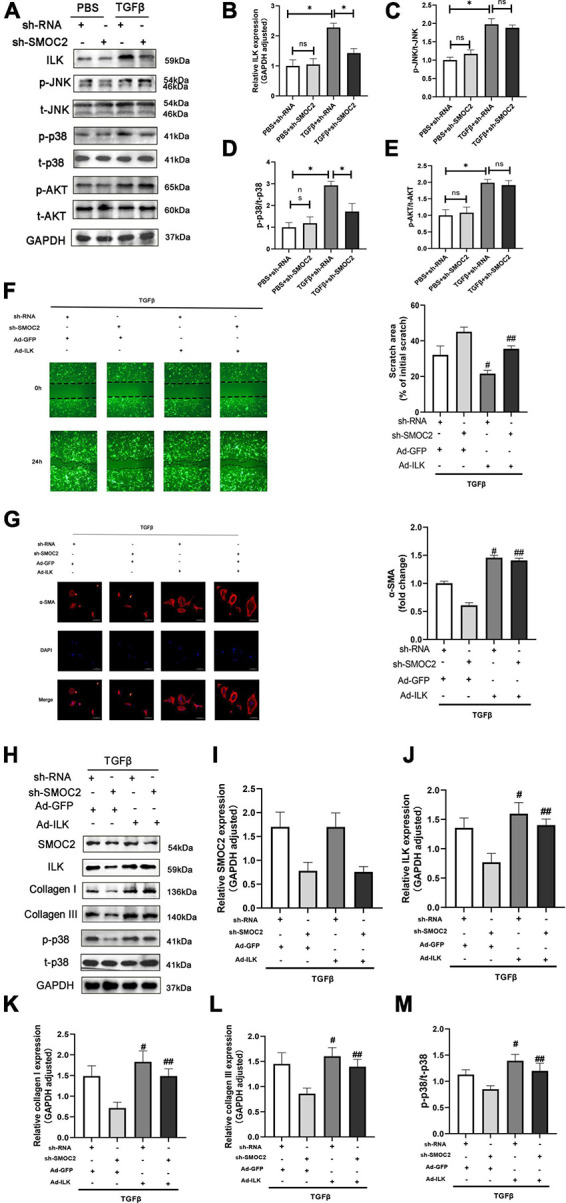
Targeting SMOC2 expression ameliorates fibrosis *via* the ILK/p38 pathway *in vitro*. **(A–E)** Representative Western blots and quantitative results of ILK, t-AKT, p-AKT, t-JNK, p-JNK, t-p38, and p-p38 (*n* = 6). *p < 0.05 vs. the control + sh-RNA group. ^#^*p* < 0.05 vs. the ISO + sh-RNA group. n.s., non-significant. **(F)** Representative images of the wound scratch assay post Ad-ILK infection. **(G)** Representative images of immunohistochemical staining for α-SMA (*n* = 4). **(H–M)** Representative Western blots and quantitative results post Ad-ILK infection (*n* = 3). *p < 0.05. n.s., non-significant. ^#^*p* < 0.05 vs. the sh-RNA + Ad-GFP group. ^##^*p* < 0.05 vs. the sh-SMOC2 + Ad-GFP group.

In order to explore the mechanisms of anti-fibrotic effects of sh-SMOC2, we also detected ILK, AKT, MAPK pathways. Consistent with the findings *in vivo*, the ILK level was elevated after being treated with TGFβ, which could be down-regulated by sh-SMOC2 administration (Figures 7A, B). In-depth studies also found that p-AKT, P-JNK, and p-P38 were significantly up-regulated, while the sh-SMOC2 infection significantly reduced p-p38 without affecting the phosphorylation levels of p-AKT and p-JNK (Figures 7A, C–E and Supplementary Figures 3A–F).

Next, we also verified whether ILK participated in the activation of p-p38 in CFs *in vitro*, and we infected CFs with Ad-ILK to overexpress ILK. In line with the above findings, the anti-fibrotic effect of sh-SMOC2 was knocked over after ILK overexpression, and the scratch experiment also suggested that ILK further promoted the migration of CFS and activated p-p38 (Figure 7F). To further confirm this notion, the expression of α-SMA by IHC staining was examined. The results also identified that Ad-ILK worsened the transformation of CFs (Figure 7G). Results of *in vitro* studies matched those of *in vivo* studies. The level of SMOC2 did not change due to overexpression of ILK (Figures 7H, I), but ILK, p-p38, collagen I and collagen III were significantly up-regulated (Figures 7H, J, K–M and Supplementary Figures 4A–D).

## Discussion

To the best of our knowledge, this is the first report on the relationship between SMOC2 and myocardial fibrosis. Our study implied that the therapeutic silencing of SMOC2 can improve ISO-induced myocardial fibrosis and heart function reduction *in vivo*, and ameliorate proliferation and collagen deposition of CFs induced by TGFβ *in vitro*, and the down-regulation of ILK/p38 may be the underlying mechanism.

In addition to being associated with unfavorable outcomes, progressive cardiac fibrosis is one of the key hallmarks of heart failure (3, 29). Several etiopathogenic mechanisms contribute to myocardial fibrosis, a complex and multifactorial condition (5, 6). At present, the treatment of myocardial fibrosis is still an intractable clinical problem that demands to be further addressed (30, 31). Currently, only a few pharmacotherapy options, such as treatments targeting the increased activity of the renin-angiotensin-aldosterone system (RAAS) and sympathetic nervous system (SNS), are available to manage cardiac remodeling and fibrosis (31, 32). Despite exhibiting excellent efficacy, in some situations, the clinical application of these drugs has been restricted due to side effects or intolerance among patients (31). A better understanding of the molecular mechanisms of cardiac fibrosis is thus essential for developing novel therapeutic approaches.

SMOC2 is an encoded modular secretion protein that is known to influence cytokine activity, destabilize cell-substrate attachment, and modulate cell differentiation and cell cycle (8, 13, 16, 33, 34). A recently published study indicates that SMOC2 is an independent prognostic marker in patients with colorectal cancers (35). SMOC2 was shown to be a promising biomarker of kidney fibrosis in patients with CKD and was able to be used by researchers as a prognostic indicator (36). Previous studies have also demonstrated that SMOC2 is highly expressed upon kidney injury or in CKD models and stimulates the production of extracellular matrix, which can be ameliorated by infecting sh-RNA targeting SMOC2 (14, 16, 36). In addition, SMOC2 also participates in pulmonary fibrosis and hepatic steatosis caused by a high-fat diet targeting TGFβ1 pathways (13, 15). However, no research has examined the potential relationship between SMOC2 and cardiac fibrosis, nor have the relevant mechanisms been explored in more depth. The present study confirms for the first time that SMOC2 may contribute to the pathogenic process of cardiac fibrosis, which could be rescued at least partially by the deletion of SMOC2 expression *in vivo* and *vitro*.

ILK, a serine-threonine-protein kinase that exerts essential effects on cell transduction and molecular scaffolding, is known as an *in vivo* target of SMOC2 (18, 19, 37). Previously accumulation of evidence identified that ILK was associated with some cardiomyopathy phenotypes, as well as involved in cardiac remodeling (37). It was revealed that ILK adaptively increased in the pressure overloaded cardiac hypertrophy model and could further participate in the activation of CFs (21, 37–39). Overexpressed ILK promotes the cardiac fibrotic process by up-regulation of nuclear factor-κB (NF-κB) in cardiac fibroblasts that activate fibrotic-related genes such as CTGF and collagen I. On the other hand, ILK knockdown by siRNA could lead to attenuation of this fibrotic action (21). Therefore, it is estimated that ILK may contribute to pathological cardiac remodeling. In line with previous studies, we found that ILK was overactivated adaptively in sustained profibrogenic factors-induced mice fibrosis model to respond to the up-regulation of SMOC2.

However, other findings provided discrepant observations. ILK may be one of the key determinants that contributed to the protective effect of myocardial re-modeling after MI during Cardiac Shock Wave Therapy (40). What’s more, ILK overexpression also minimized cardiac remodeling and improved reperfusion after ischemia (41). And these were in agreement with the corresponding findings of another study (42). Researchers have recently published a study showing that targeting the regulatory ILK and relevant pathways ameliorates cardiac function in dilated myocardial animals induced by DOX, strongly implicating increased expression of ILK may ameliorate the prognosis in patients with HF (43). A mouse model of the ILK knockout was also shown to alter myocardial electrical properties, potentially resulting in fatal arrhythmias (37). Taken together, these studies suggest an intricate relationship between ILK and cardiac fibrosis and remodeling, and future research is still desperately warranted to shed light on this issue.

Evidence is mounting that the MAPK pathway, such as p38 and JNK1/2, functions in regulating cardiac apoptosis, hypertrophy, and fibrosis (44–47). And p38 was thought to be a dominant regulator in myocardial fibrosis (48). Additionally, it is interesting to note that several studies have identified the AKT pathway as being associated with fibrosis of the heart and collagen synthesis (5). The results of our present study are consistent with the previous observations, showing that AKT, p38, and JNK1/2 MAPKs were slightly activated following the pro-fibrotic stimuli *in vivo* and *vitro*. However, unexpectedly, SMOC2 knockdown impaired the expression of p38 without affecting AKT or JNK1/2 pathways, revealing p38 as a contributor to the development of SMOC2-mediated cardiac fibrosis. It was well-established that p38 phosphorylation in CFs could drive myofibroblasts to synthesize and formate collagen, which could be reversed when p38 was inhibited (6, 45). P38 has been implicated in a variety of pathogenetic processes: such as inflammation (49), osteogenic differentiation (50), apoptosis (51), and cancer differentiation (52). Specifically, p38 could be activated by several regulators thus exerting its physiological effects as a result. Zhang et al. (6) discovered that p38 was mediated by ribosomal protein S5 (RPS5) in press overload-induced cardiac fibrosis mice, whereas matrine administration alleviated this process. Additionally, Meijles et al. (53) revealed that p38 was regulated by apoptosis signal-regulating kinase 1 (AKS1) during the process of fibrosis and subsequent deterioration of hypertensive heart disease.

Interestingly, in a study, scholars found that ILK/p38 MAPK pathway can regulate the osteogenic effect on the surface of the a-C coated titanium (24). They found that ILK was significantly up-regulated on the surface of the C-Ti, and siRNA targeting ILK reduced p38 phosphorylation and osteogenic differentiation in the C-Ti surface, which shed light on the research into the relationship between ILK and p38 in the present study. As anticipated, overexpression of ILK *in vivo* or *vitro* would result in increased phosphorylation of p38 MAPK accompanied by elevated markers of cardiac fibrosis.

## Conclusion and limitations

**FIGURE 8:**
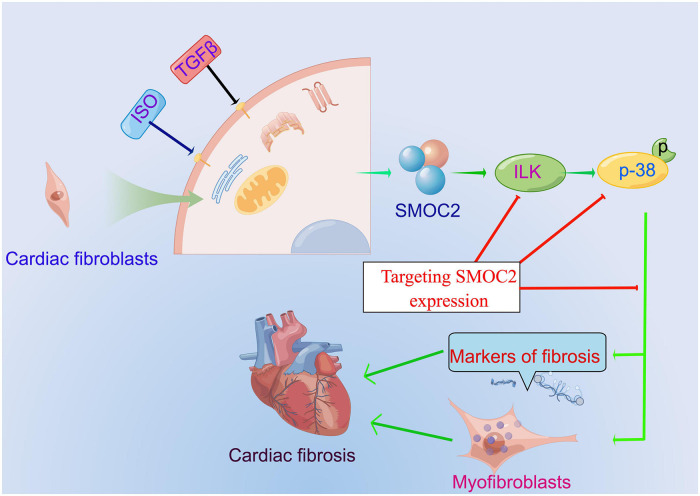
The proposed working model of targeting SMOC2 expression on pathological cardiac fibrosis.

Our study identified that SMOC2 is involved in collagen deposition, cardiac fibroblasts activation, and, ultimately, cardiac fibrosis for the first time in response to pro-fibrotic stimuli such as ISO and TGFβ and ILK/p38 signaling pathway may be responsible for this important process (Figure 8). Furthermore, therapeutic SMOC2 silencing has protective effects against cardiac fibrosis by inhibiting ILK/p38 signaling, validating the critical role of SMOC2 in mediating cardiac fibrosis, and providing promising prevention and control strategies for cardiac remodeling management in clinical practice.

There are several limitations to this study, despite its promising results. Firstly, we only applied the cardiac fibrosis model induced by ISO, and did not evaluate other cardiac fibrosis models. Secondly, a human validation of the findings was not performed. Moreover, an important limitation to this study is the use of neonatal cardiac fibroblasts. Finally, this study did not address the role of SMOC2 in cardiomyocytes and its role in cardiac hypertrophy remains to be defined. Therefore, the results of the study should be interpreted with caution.

## Data availability statement

The original contributions presented in this study are included in the article/Supplementary material, further inquiries can be directed to the corresponding author.

## Ethics statement

The animal study was reviewed and approved by the Animal Care and Use Committee of Renmin Hospital of Wuhan University.

## Author contributions

HR and FZ performed the experiments and analyzed the data. HR, LY, and JH designed the experiments, supervised and conceptualized the study, and wrote and edited the manuscript. HR and FZ wrote and edited the manuscript. All authors contributed to the article and approved the submitted version.

## Supplementary material

Supplementary Figure 1 Total and phosphorylated protein levels *in vivo*. **(A–F)** Quantitative results of t-AKT, p-AKT, t-JNK, p-JNK, t-p38, and p-p38 (*n* = 6). **p* < 0.05. n.s., non-significant.

Supplementary Figure 2 Collagen I **(A)** and III **(B)** content in the cell culture supernatant. **p* < 0.05. n.s., non-significant.

Supplementary Figure 3 Total and phosphorylated protein levels *in vitro*. **(A–F)** Quantitative results of t-AKT, p-AKT, t-JNK, p-JNK, t-p38, and p-p38 (*n* = 6). **p* < 0.05. n.s., non-significant.

Supplementary Figure 4 Collagen I **(C)** and III **(D)** content in the cell culture supernatant and total **(A)** and phosphorylated **(B)** protein levels of p-38. n.s., non-significant. ^#^*p* < 0.05 vs. the sh-RNA+Ad-GFP group. ^##^*p* < 0.05 vs. the sh-SMOC2+Ad-GFP group.

Supplementary Figure 5 Original band of western blot in Figure 1A.

Supplementary Figure 6 Original band of western blot in Figure 2A.

Supplementary Figure 7 Original band of western blot in Figure 3C.

Supplementary Figure 8 Original band of western blot in Figure 4A.

Supplementary Figure 9 Original band of western blot in Figures 5A,B.

Supplementary Figure 10 Original band of western blot in Figure 6A.

Supplementary Figure 11 Original band of western blot in Figure 6J.

Supplementary Figure 12 Original band of western blot in Figure 7A.

Supplementary Figure 13 Original band of western blot in Figure 1H.
