# Supplementary figures and images for: Suppression of SMOC2 alleviates myocardial fibrosis via the ILK/p38 pathway
Source: Front Cardiovasc Med. 2023 Mar 2;9:951704. doi: 10.3389/fcvm.2022.951704 (PMC10017443; doi:10.3389/fcvm.2022.951704)

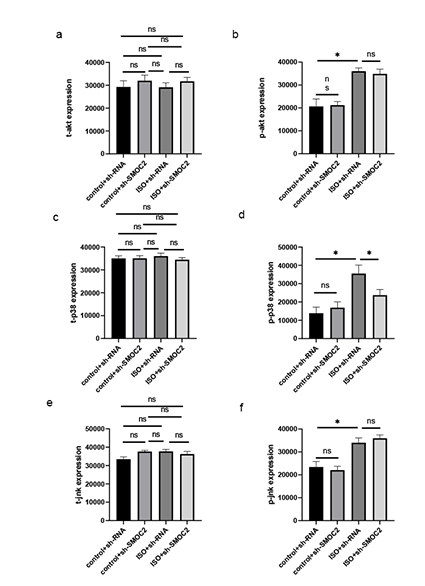

Supplement: Supplementary Figure 1 — Total and phosphorylated protein levels in vivo. (A–F) Quantitative results of t-AKT, p-AKT, t-JNK, p-JNK, t-p38, and p-p38 (n = 6). *p < 0.05. n.s., non-significant. [file Image_1.TIF]

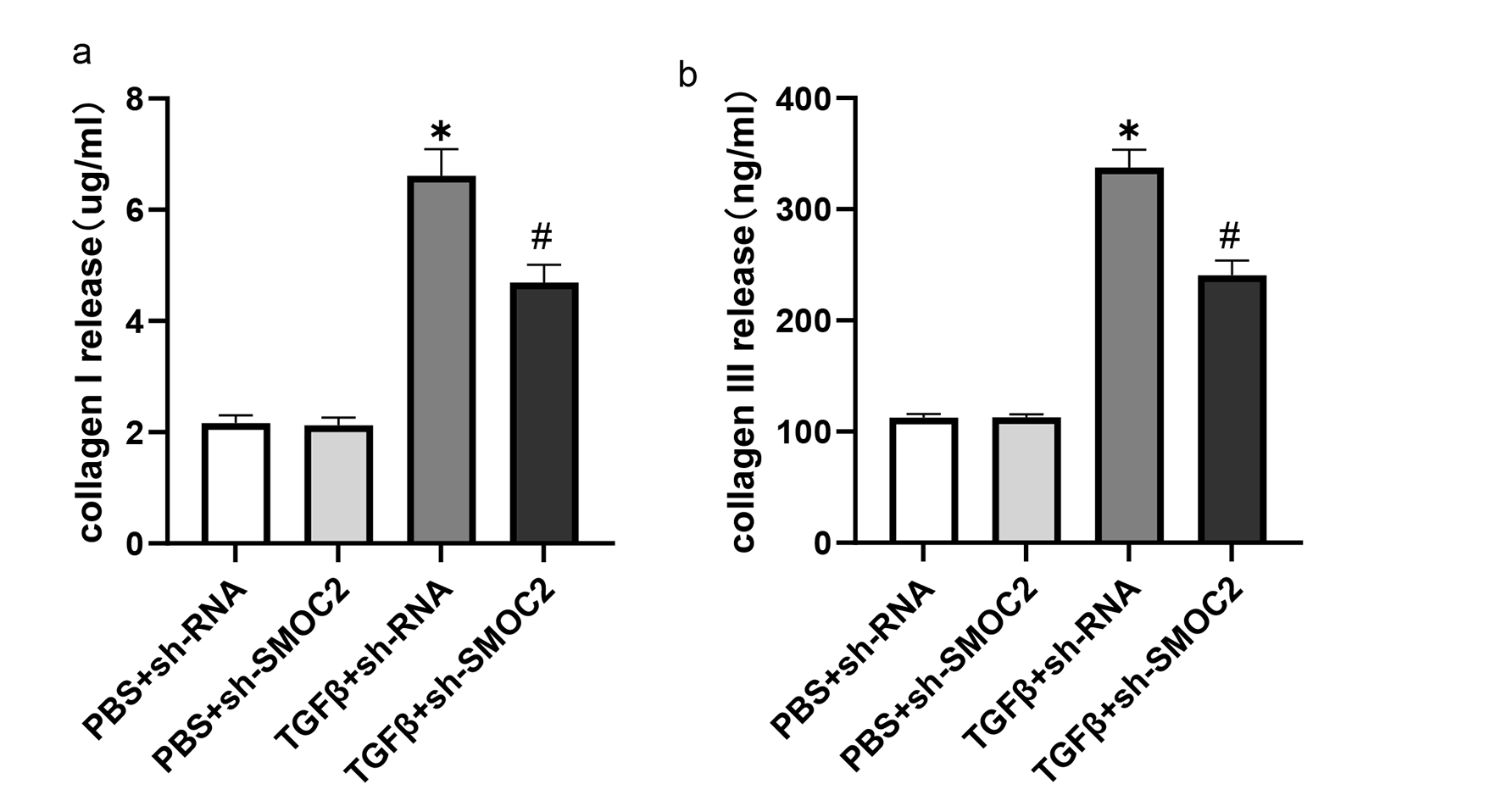

Supplement: Supplementary Figure 2 — Collagen I (A) and III (B) content in the cell culture supernatant. *p < 0.05. n.s., non-significant. [file Image_2.TIF]

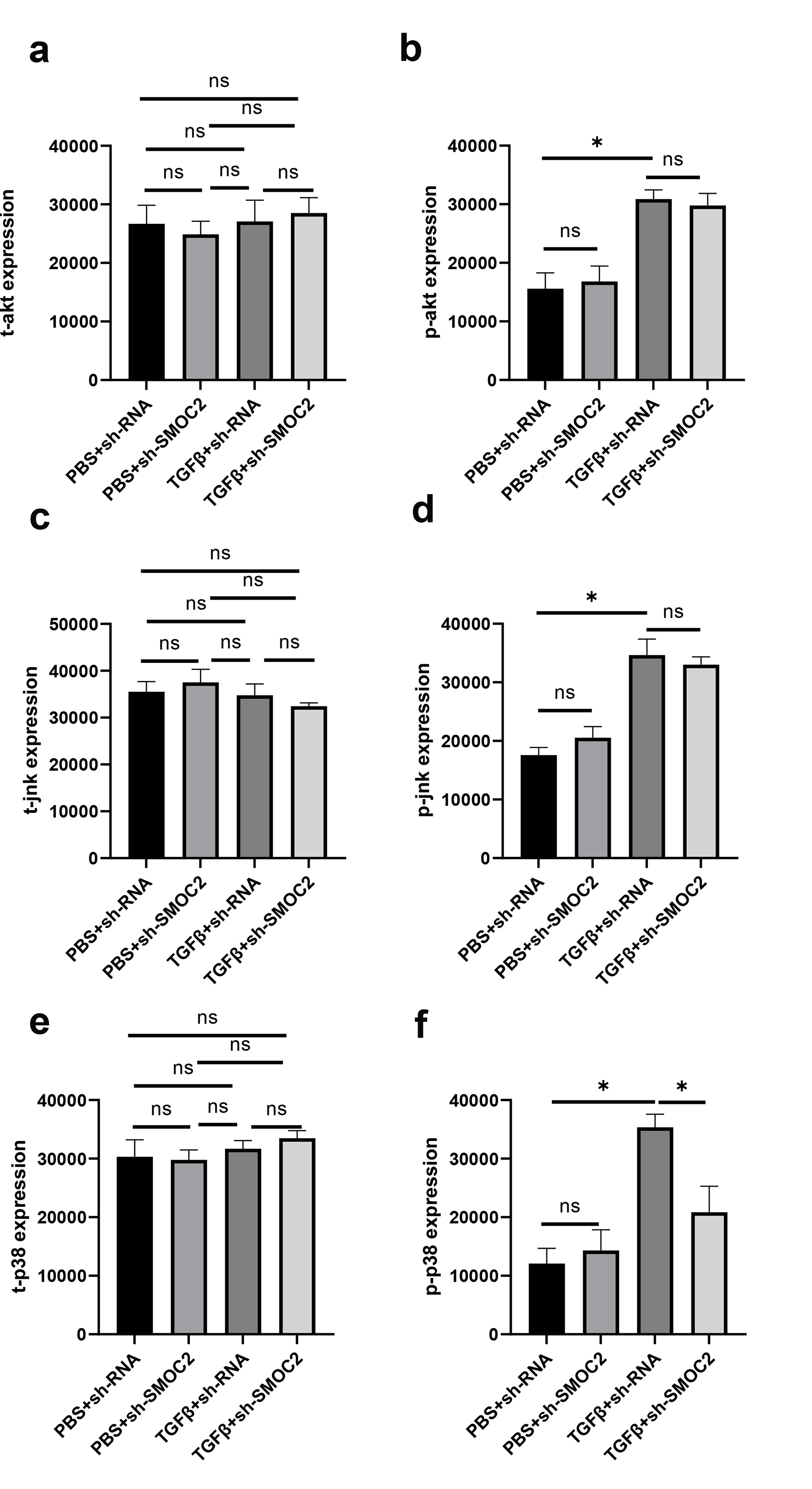

Supplement: Supplementary Figure 3 — Total and phosphorylated protein levels in vitro. (A–F) Quantitative results of t-AKT, p-AKT, t-JNK, p-JNK, t-p38, and p-p38 (n = 6). *p < 0.05. n.s., non-significant. [file Image_3.TIF]

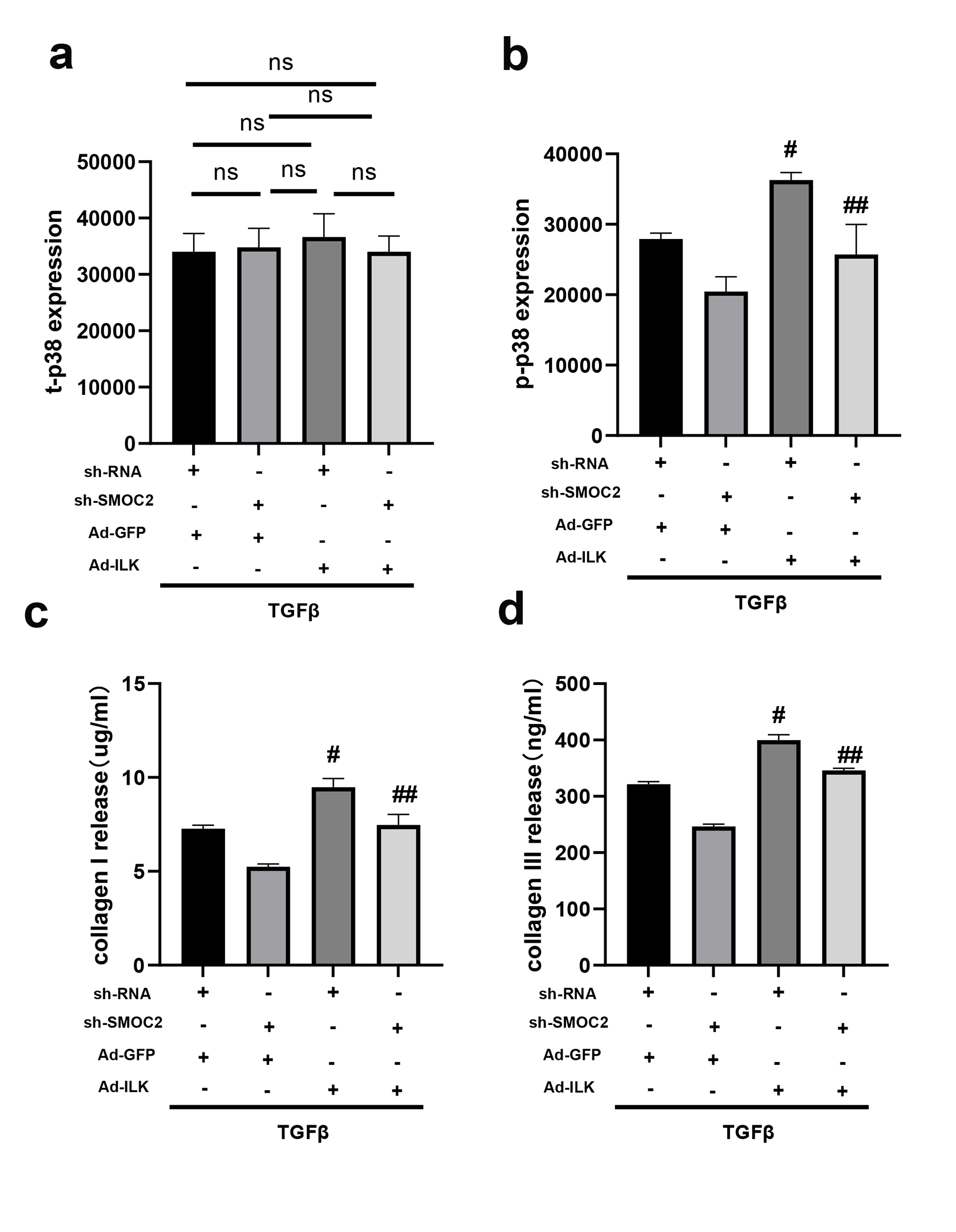

Supplement: Supplementary Figure 4 — Collagen I (C) and III (D) content in the cell culture supernatant and total (A) and phosphorylated (B) protein levels of p-38. n.s., non-significant. #p < 0.05 vs. the sh-RNA+Ad-GFP group. ##p < 0.05 vs. the sh-SMOC2+Ad-GFP group. [file Image_4.TIF]

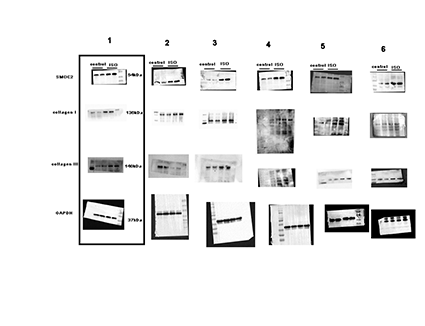

Supplement: Supplementary Figure 5 — Original band of western blot in Figure 1A. [file Image_5.TIF]

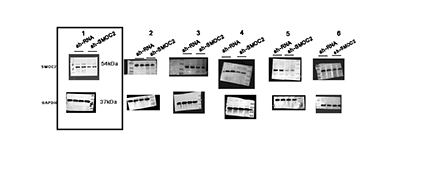

Supplement: Supplementary Figure 6 — Original band of western blot in Figure 2A. [file Image_6.TIF]

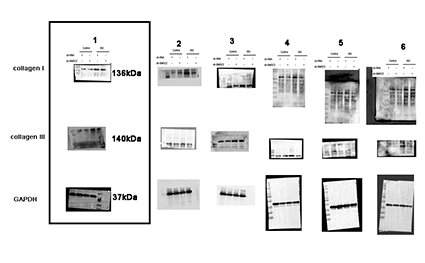

Supplement: Supplementary Figure 7 — Original band of western blot in Figure 3C. [file Image_7.TIF]

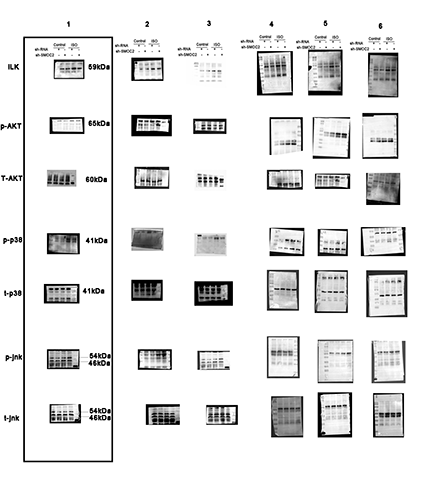

Supplement: Supplementary Figure 8 — Original band of western blot in Figure 4A. [file Image_8.TIF]

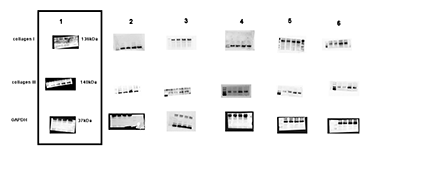

Supplement: Supplementary Figure 9 — Original band of western blot in Figures 5A,B. [file Image_9.TIF]

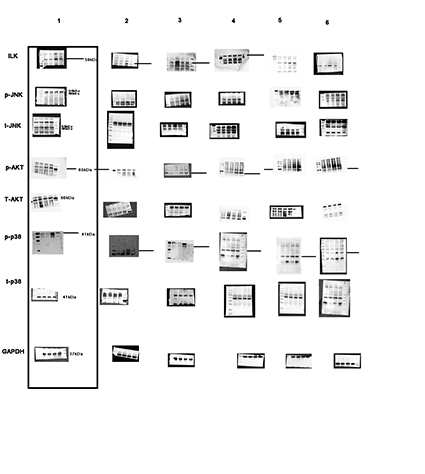

Supplement: Supplementary Figure 10 — Original band of western blot in Figure 6A. [file Image_10.TIF]

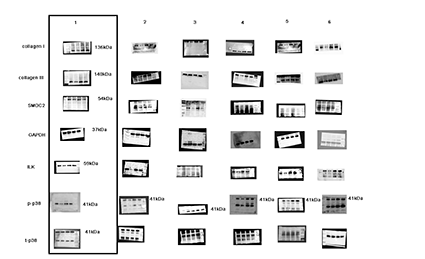

Supplement: Supplementary Figure 11 — Original band of western blot in Figure 6J. [file Image_11.TIF]

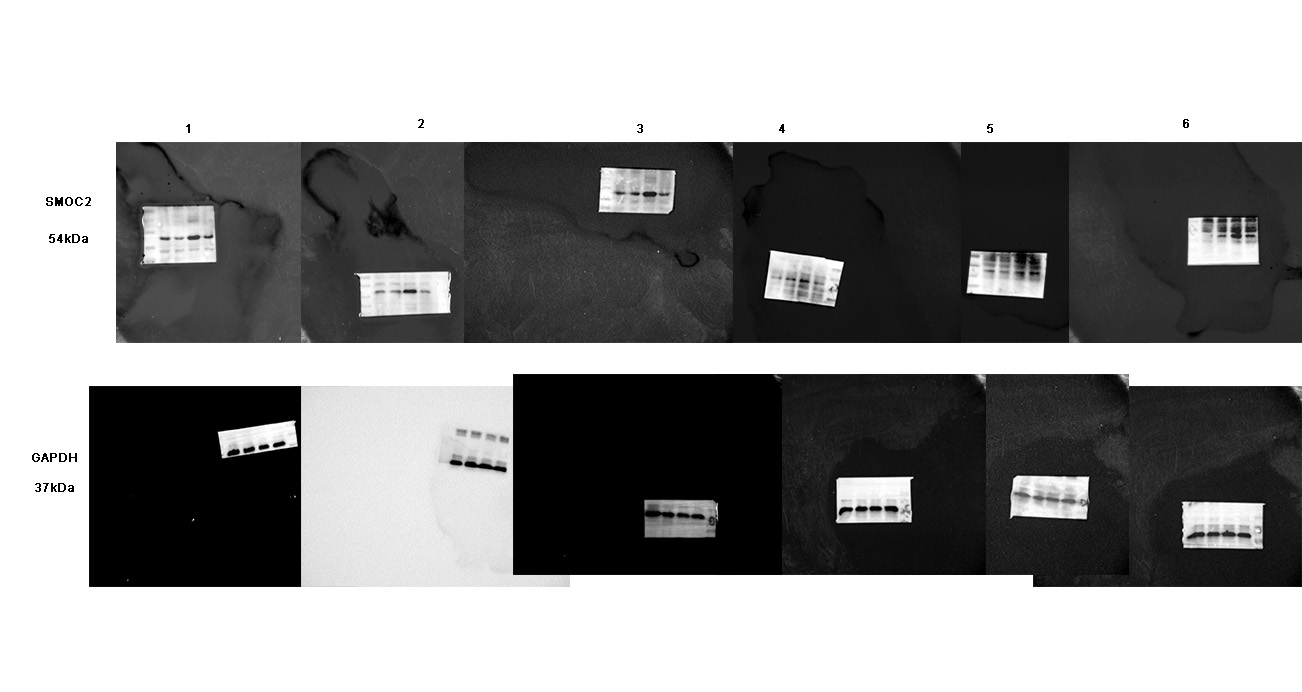

Supplement: Supplementary Figure 12 — Original band of western blot in Figure 7A. [file Image_12.JPEG]

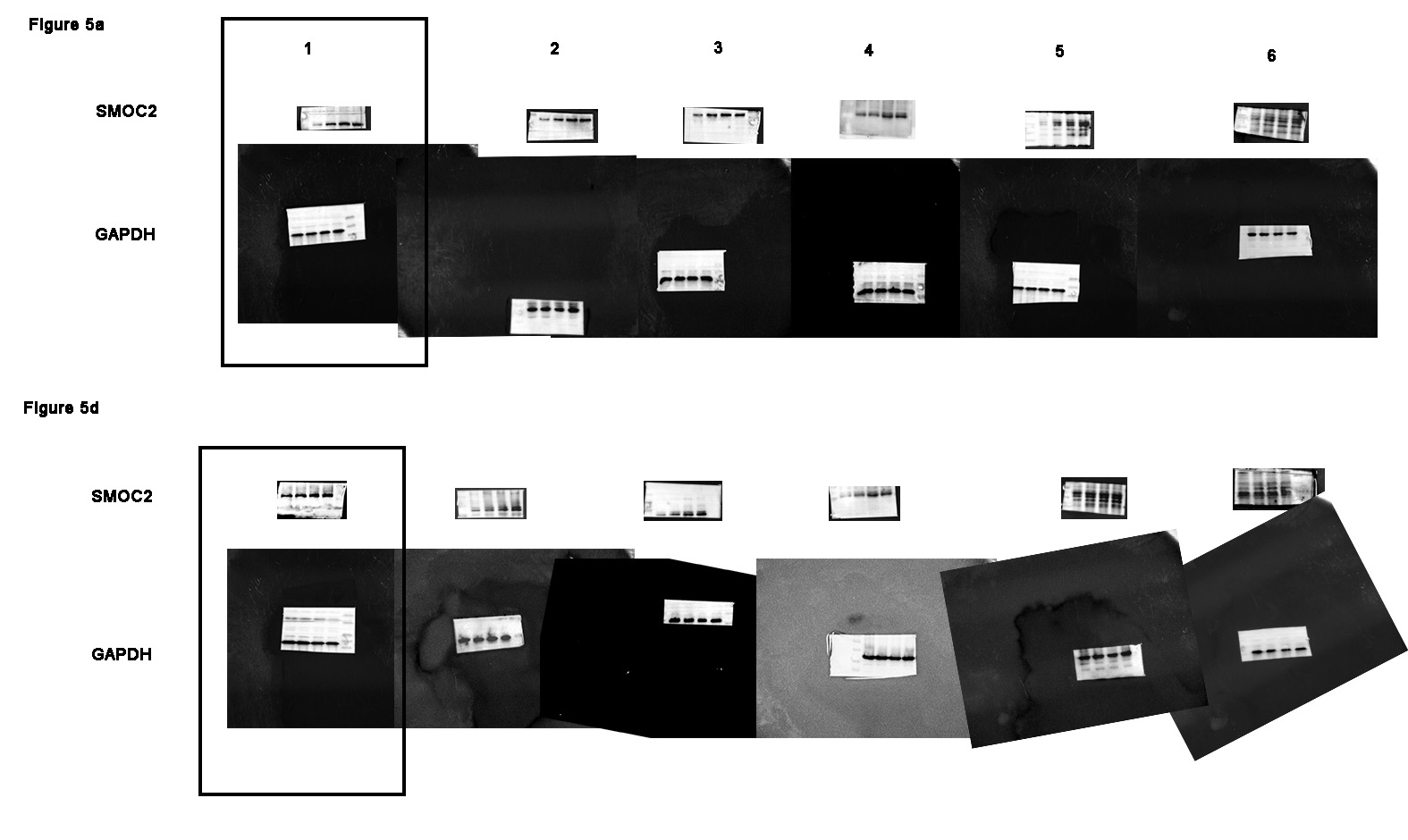

Supplement: Supplementary Figure 13 — Original band of western blot in Figure 1H. [file Image_13.JPEG]
